# Supplementary figures and images for: ‘Not only faces’: specialized visual representation of human hands revealed by adaptation
Source: R Soc Open Sci. 2020 Dec 16;7(12):200948. doi: 10.1098/rsos.200948 (PMC7813241; doi:10.1098/rsos.200948)

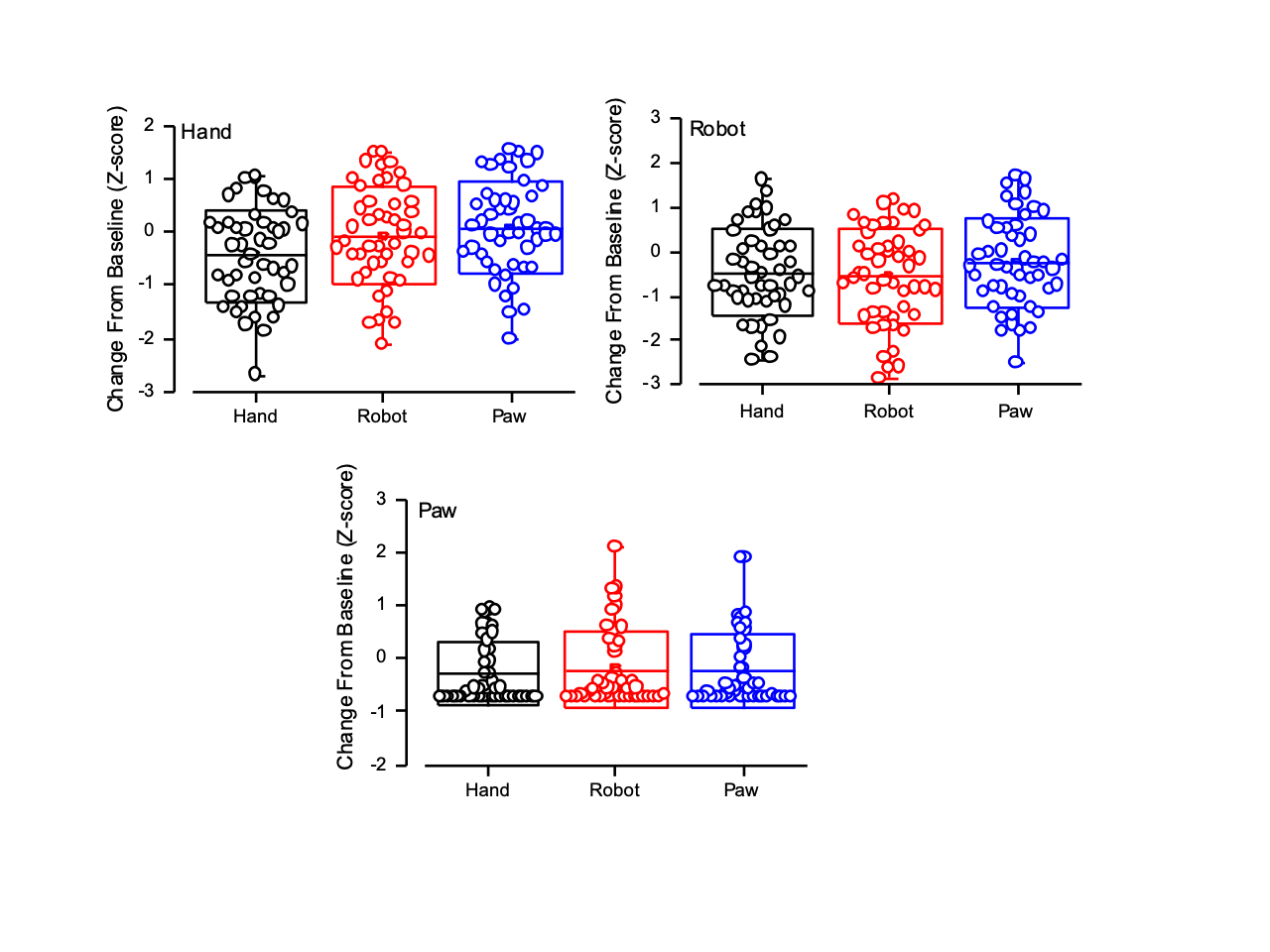

Supplement: supplementary figure [file rsos200948supp1.tiff]
